# Effect of Additive Removal on the Physicochemical Properties of Gluten-Free Bread

**DOI:** 10.3390/foods15020338

**Published:** 2026-01-16

**Authors:** Ramón Torres-Pérez, Marta Maravilla Siguero-Tudela, Tania Doménech, Purificación García-Segovia, Javier Martínez-Monzó, Marta Igual

**Affiliations:** i-Food, Instituto Universitario de Ingeniería de Alimentos-FoodUPV, Universitat Politècnica de València, Camino de Vera s/n, 46022 Valencia, Spain; ramon@sinblat.es (R.T.-P.); martamaravilla00@gmail.com (M.M.S.-T.); tdomval@doctor.upv.es (T.D.); pugarse@tal.upv.es (P.G.-S.); xmartine@tal.upv.es (J.M.-M.)

**Keywords:** gluten-free bread, clean label, leavening agents, monocalcium phosphate, sodium bicarbonate, mono- and diglycerides, crumb texture, staling

## Abstract

The growing demand for clean-label gluten-free bread is driving a reduction in additives, although their technological roles are not yet fully understood. This study evaluated the effect of progressively removing monocalcium phosphate, sodium bicarbonate, and mono- and diglycerides (MDG) on the quality of gluten-free bread during storage. Four formulations were prepared: a reference (RF) containing all additives, and three reduced-additive versions without monocalcium phosphate (FA), without monocalcium phosphate and sodium bicarbonate (FB), or without any additives (FC). Specific volume, moisture, water activity, crumb structure, color, and texture were assessed on days 1, 8, 15, and 22. Additive removal significantly affected bread quality: the formulation without leavening agents (FB) showed the lowest specific volume (≈2.8 cm^3^/g) and the highest crumb hardness (≈38 N), whereas the additive-free formulation (FC) achieved the highest specific volume (≈3.3 cm^3^/g) and a crumb structure comparable to the reference bread, with a higher void fraction (≈28%). During storage, all breads exhibited increasing hardness, although FC did not stale faster than RF, likely due to its higher specific volume after baking. The results confirm that sodium bicarbonate and monocalcium phosphate are essential for gas generation and structural development, while removal of MDG improved loaf volume without intensifying deterioration.

## 1. Introduction

In recent years, the demand for gluten-free bread has experienced significant growth, driven both by the increasing prevalence of celiac disease and the general trend towards healthier diets and functional food products [1,2]. This growing interest has encouraged both the food industry and academic research to develop gluten-free formulations capable of achieving products with quality characteristics like those of conventional wheat bread. However, the absence of gluten poses important technological challenges, especially regarding the structure, texture, and acceptability of the final product [3]. In this context, it becomes necessary to explore new ingredients and technological strategies to improve the quality of gluten-free bread.

Moreover, this technological challenge is intensified by consumers’ increasing concern regarding food composition, driving a strong trend towards cleaner-label products with fewer additives. Currently, the demand for cleaner and more natural formulations has led the baking industry, particularly the gluten-free sector, to reconsider the use of additives. Recent studies indicate that consumers show a clear preference for products with fewer additives, seeking more natural alternatives without compromising quality [4,5,6]. This trend has prompted the reformulation of products by reducing or eliminating ingredients such as emulsifiers, dough improvers, or chemical leavening agents, despite the challenges this poses in terms of volume, texture, and stability. In this regard, several authors have highlighted that the “clean-label” trend has also reached the gluten-free breadmaking sector, where the removal of additives presents significant challenges to maintaining the technological properties of the final product [7,8]. Nevertheless, consumers’ positive acceptance of these products has promoted research focused on developing gluten-free formulations that reduce or substitute additives without compromising bread quality [9].

In gluten-free breadmaking, the combination of sodium bicarbonate and leavening acids, such as monocalcium phosphate or acid pyrophosphate, is one of the most common strategies for generating carbon dioxide, thus facilitating dough expansion and the formation of the characteristic bread crumb structure [10]. In fact, recent studies indicate that approximately 19% of commercial gluten-free bread formulations include sodium bicarbonate and around 7% incorporate typical leavening acids [11]. The controlled generation of carbon dioxide not only allows for dough expansion but also directly affects the final texture and volume of the bread by determining the size and distribution of gas bubbles [12]. Monocalcium phosphate was the first phosphate used as a leavening agent in baking powders, patented by Horsford in 1856 [13]. Although calcium phosphates are considered safe food additives and have been routinely used for decades [14], recent studies have associated them with vascular damage and increased mortality, particularly in individuals with renal or cardiovascular disease [15].

From a physicochemical perspective, mono- and diglycerides of fatty acids (MDG) act as emulsifiers due to the polar duality of their molecular structure [16], and are widely used in breadmaking due to their ability to improve texture and extend bread shelf life [17,18]. These additives are present in approximately 28% of commercial gluten-free bread formulations [11]. Their main mechanism of action is related to their capacity to form inclusion complexes with amylose, limiting starch retrogradation and thereby contributing to the maintenance of crumb softness during storage [19].

Monoglycerides are widely used as emulsifiers in bakery systems due to their ability to interact with starch and proteins. They can form amylose–lipid complexes that modify starch gelatinization and thermal properties in baked products [20,21,22], and have been reported to interact with proteins such as β-lactoglobulin to form structured complexes that influence starch–protein organization [23]. In wheat-based systems, monoglycerides can bind to gluten proteins, enhancing protein–protein interactions through reduced electrostatic repulsion and increased hydrophilic and hydrophobic interactions, which can affect dough strength [24]. In gluten-free formulations, these interactions may produce technological effects that are highly dependent on the matrix and formulation. For example, Jyotsna et al. [25] observed increased volume in gluten-free muffins when hydroxypropyl methylcellulose (HPMC) was combined with monoglycerides, indicating potential synergistic effects with structuring agents. However, in gluten-free bread, monoglycerides alone have been reported to reduce loaf volume and specific volume, whereas combinations with other emulsifiers such as SSL or DATEM can increase these parameters [26]. Within this framework, the present research aimed to investigate the effect of progressively removing key additives commonly used in gluten-free bread formulations on the physical, structural, and textural characteristics of the final product.

To address these challenges, this study systematically evaluated the impact of progressive additive removal on the quality and staling behavior of gluten-free bread. Four experimental formulations were developed, including a control formulation containing all the additives under study (monocalcium phosphate, sodium bicarbonate, and mono- and diglycerides of fatty acids), representative of commercial gluten-free bread products. A comprehensive characterization of the bread’s physical, structural, and textural attributes was conducted, including an analysis of the pasting properties of flour blends using a Rapid Visco Analyzer (RVA) and a detailed monitoring of quality changes during storage (days 1, 8, 15, and 22). By addressing the technological challenges of additive removal, this study provides critical insights into the feasibility of producing cleaner-label gluten-free bread while ensuring consumer acceptance and shelf-life stability.

## 2. Materials and Methods

### 2.1. Formulations and Ingredients

Gluten-free bread was prepared using sunflower oil (Aceites La Canal, Chella, Spain), rice syrup (Ferrer Alimentación, Barcelona, Spain), compressed yeast (Lesaffre Ibérica, Valladolid, Spain), and a gluten-free baking mix (Sinblat Alimentación Saludable, Foios, Spain), which was composed of corn starch, rice flour, tapioca starch, salt, hydroxypropyl methylcellulose (HPMC), psyllium fiber, bamboo fiber, xanthan gum, pea protein, potassium sorbate, and calcium propionate.

The additives used in this study were as follows: Monocalcium phosphate monohydrate (Ca(H_2_PO_4_)_2_·H_2_O; E341(i)), used as an acidifying agent, was supplied by Emilio Peña, S.A. (Torrent, Valencia, Spain), sodium bicarbonate (EPSA, Torrente, Spain), and Mono- and diglycerides of fatty acids (E471), supplied by Brenntag Química S. A. U. (Dos Hermanas, Seville, Spain), were used as an emulsifier in this study. According to the manufacturer’s specifications, the emulsifier contained > 95% monoester, predominantly composed of C16:0 (palmitic acid) and C18:0 (stearic acid), derived from palm oil, with a melting point of 63–68 °C.

The gluten-free bread recipe was composed of (per 100 g of gluten-free baking mix): 112.5 g of water, 19.7 g of sunflower oil, 3.9 g of rice syrup, 3.7 g of sugar, and 3.7 g of yeast.

To evaluate the impact of progressive additive removal on gluten-free bread quality, four experimental formulations were developed (Table 1). The Reference Formulation (RF) contained monocalcium phosphate, sodium bicarbonate, and mono- and diglycerides of fatty acids, as a control formulation commonly found in gluten-free bread products. From this base, three modified formulations were prepared: Formulation A (FA), where monocalcium phosphate was removed; Formulation B (FB), where both monocalcium phosphate and sodium bicarbonate were excluded; and Formulation C (FC), where mono- and diglycerides of fatty acids were also eliminated, resulting in a bread free from these three additives. This sequential removal allowed for a systematic assessment of how each additive contributes to the physical, structural, and textural properties of the final product.

### 2.2. Dough Preparation and Baking Process

All the ingredients were mixed using a Sigma Aeromix mixer (Sigma SRL, Torbole Casaglia, Italy) with the hook attachment at speed one for 15 min, as previously described by Torres-Pérez et al. [27]. The dough was then divided into 440 g portions and shaped by hand before being placed into a Teflon-coated metal mold measuring 9.9 × 19.1 × 6.8 cm. These pieces were fermented in a Bauuman fermenter (Bauuman Tech. SL, Valencia, Spain) at 29 °C and 80% relative humidity until they tripled in volume.

Each bread formulation was prepared in duplicate (*n* = 2) to ensure reproducibility of the results. After fermentation, the bread was placed in an oven (FOX 10T-LFRC, Logiudice Forni SRL Unipersonale, Arcole, Italy) and baked at 210 °C for 2 min, followed by 38 min at 180 °C. After baking, the loaves were removed from the molds and left to cool for 2 h at 25 °C. Once cooled, the loaves were weighed, and their specific volume was measured. Subsequently, the breads were sliced, packed in polypropylene bags, and stored under controlled conditions (22–25 °C, 50–70% RH).

### 2.3. Pasting Properties (RVA Analysis)

Pasting behavior was evaluated using 3.5 g (±0.1 g) of each formulation described in Table 1 and 25 g (±0.1 g) of distilled water. The standard RVA profile was applied, consisting of an initial holding stage at 50 °C for 1 min, followed by a heating phase up to 95 °C, a holding period at this temperature for 2 min and 30 s, and a cooling phase down to 50 °C with a final holding period of 4 min, in accordance with method 61-02.01 [28]. The following pasting parameters were recorded: peak viscosity, trough viscosity, breakdown, setback, final viscosity, and pasting temperature. All measurements were performed in duplicate.

### 2.4. Physicochemical Analysis

The following physicochemical analyses were performed on the breads obtained from each experimental formulation.

#### 2.4.1. Baking Loss

Baking loss was determined following the method described by Oliveira et al. [29], using a digital scale with a precision of 0.01 g. Measurements were performed on twenty loaves per formulation before slicing and packaging. The results were expressed as the percentage of weight loss relative to the initial dough weight (%).

#### 2.4.2. Specific Volume

The specific volume of the bread was determined according to the AACC (2010) method [30], using chia seeds as the displacement material. Measurements were performed on twenty loaves per formulation before slicing and packaging. The specific volume was calculated as the ratio between bread volume and its weight (cm^3^/g).

#### 2.4.3. Moisture Content

The moisture content (MC) of the samples was determined by vacuum drying using a Vaciotem-T vacuum oven (J.P. Selecta, S.A., Barcelona, Spain). Samples were pre-dried at 40 °C for 24 h and subsequently dried in the vacuum oven at 70 ± 1 °C under reduced pressure (<100 mmHg) for 48 h, until constant weight, following the method described by [31]. The determinations were carried out the day after baking, using the central slices of each bread loaf. Measurements were performed in duplicate.

#### 2.4.4. Water Activity

Water activity (aw) was measured using an AquaLab PRE device (Decagon Devices Inc., Pullman, WA, USA). Measurements were performed at a controlled temperature according to the manufacturer’s instructions. The determinations were carried out in duplicate using the central slices of each bread loaf.

#### 2.4.5. Crumb Image Analysis

Digital images of 10 mm thick bread slices were acquired using an Epson SX420 scanner (Seiko Epson Corporation, Tokyo, Japan). Image contrast and brightness were adjusted to +50% and +80%, respectively, using Windows Photo Viewer software (Windows 11). Subsequently, the images were processed and analyzed using ImageJ software (version 1.41; NIH, Bethesda, MD, USA).

For the analysis, the central area of each slice was cropped to a 5 cm × 5 cm field of view. In each image, the total number of cells and the average cell area (in mm^2^) were determined. Cells were defined as structures with an area ranging from 0.15 to 10.00 mm^2^, following the criteria proposed by Sciarini et al. [32]. Based on these data, the proportions of small cells (0.15 < area < 4.00 mm^2^) and large cells (4.00 < area < 10.00 mm^2^) were calculated. Image analysis was performed on six slices per formulation, obtained from different loaves.

#### 2.4.6. Color Analysis

Color analysis was performed on the crusts and crumbs of the breads obtained from each formulation on storage days 1, 8, 15, and 22. Measurements were carried out using a CR-400 colorimeter (Konica Minolta, Tokyo, Japan), calibrated with the standard D65 illuminant and a 10° standard observer, following the CIELab color space.

The evaluated parameters were L* (lightness), a* (green–red axis), and b* (blue–yellow axis). To observe the color changes between samples, the total color difference (ΔE*) was calculated according to the following formula:

ΔE* = [(ΔL*)^2^ + (Δa*)^2^ + (Δb*)^2^]^½^ (1)

The parameter ΔE_1_* is indicative of the color changes that occur between the reference formulation (RF) and the FA, FB, and FCs on day 1.

The parameters ΔL*, Δa*, Δb*, and ΔE_2_* were determined during the storage period. The color variations that occurred were calculated for each formulation, evaluating the difference between each storage period and the initial color. All measurements were performed on crust and crumb, with six replicates per formulation per storage day.

### 2.5. Texture Analysis

Texture analyses of crumb and crust were performed on days 1, 8, 15, and 22 of storage.

Crumb texture was evaluated through a Texture Profile Analysis (TPA) using a TA-XT Plus texture analyzer equipped with a 50 kg load cell (Stable Micro Systems, Surrey, UK). A P/75 compression plate was used, with a test speed of 5 mm/s and a deformation of 50%. For each formulation, eight central slices (10 mm thick) were analyzed, considering each slice as an independent replicate.

The parameters assessed were hardness, cohesiveness, springiness, chewiness, and gumminess, which were calculated from the force–time curve generated by the characteristic double-compression cycle of the TPA test [33].

Crust texture was analyzed using a puncture test. Rectangular samples of 1 cm in length and 0.5 cm in thickness were prepared. This test determines the maximum puncture force, defined as the force required to insert a metal probe into the sample, providing a measure of crust firmness and cohesiveness.

A stainless-steel needle probe (P/2N) was used, and the test parameters were as follows: pre-test speed of 0.6 mm/s, test speed of 0.6 mm/s, post-test speed of 10 mm/s, and a displacement of 20 mm. The maximum puncture force, expressed in Newtons, was recorded as the response variable. Eight replicates per formulation and per storage day were performed.

### 2.6. Statistical Analyses

The data were subjected to analysis of variance (ANOVA), and mean comparisons were performed using Fisher’s Least Significant Difference (LSD) test at a 95% confidence level (*p* < 0.05). All statistical analyses were conducted using Statgraphics Centurion 19 v19.1.2 (Statgraphics Technologies, Inc., The Plains, VA, USA).

## 3. Results and Discussion

The results obtained from the physicochemical and functional analyses of the bread formulations are presented and discussed below, following the structure of the experimental procedures.

### 3.1. Effect of Additive Removal

The removal of functional additives commonly used in gluten-free bread formulations can significantly affect the dough behavior during processing and the quality attributes of the final product. This section presents the results obtained from the different formulations—RF (reference), FA, FB, and FC—focusing on their impact on the pasting behavior, physicochemical properties, internal crumb structure, and color at day 1 of storage.

#### 3.1.1. Effect of Additive Removal on Pasting Properties

Results of viscosity properties are presented in Table 2. The pasting temperature showed significant differences among formulations (*p* < 0.05), with the lowest value observed in FC (50.2 ± 0.1 °C), in contrast to the higher and similar values recorded for RF, FA, and FB (~73.5–73.8 °C).

The time to peak viscosity did not differ significantly between formulations, with values ranging from 4.8 to 5.1 min. Similarly, peak viscosity (3907–4186 cP) was not significantly affected by the removal of additives.

In contrast, significant differences were observed in the minimum viscosity (trough) during the high-temperature holding stage. Since all formulations exhibited similar peak viscosities, these differences in the trough were directly reflected in the breakdown (defined as the difference between peak and trough viscosity). The FA formulation (without monocalcium phosphate) showed significantly different values for both parameters compared to the reference RF, while FB and FC presented intermediate values, with no significant differences among themselves or with RF.

Finally, final viscosity values (3025.5–3658.5 cP) and setback viscosity did not vary significantly among formulations.

In the present study, the removal of monocalcium phosphate in the FA formulation resulted in a significant increase in trough viscosity and, consequently, a reduction in breakdown. This is because breakdown is calculated as the difference between peak viscosity and trough viscosity; thus, an increase in the latter parameter directly leads to a decrease in breakdown. Breakdown reflects the disintegration of the gelatinized starch granule structure during prolonged agitation and heating [34]. In general terms, a higher breakdown value indicates a lower capacity of the starch matrix to withstand heat and shear stress during thermal processing [35].

In a study conducted by Zhou et al. [36], it was observed that phosphates modify starch gelatinization during thermal processing. The authors reported that RVA parameters, such as peak viscosity, trough viscosity, and breakdown, vary depending on the type and concentration of phosphate used. These changes are related to the effect of phosphates on the pH of the system and their interaction with starch granules during heating.

In our study, the removal of monocalcium phosphate in the FA formulation may be related to a change in the acid-base balance of the system. Monocalcium phosphate acts as an acidifying agent in the presence of sodium bicarbonate [13], neutralizing its alkalizing effect. The absence of this phosphate in FA, and consequently the lack of bicarbonate neutralization, could explain the observed increase in trough viscosity and reduction in breakdown, although the pH of the mixtures was not directly measured.

However, these results partially contrast with previous studies, which demonstrate that alkaline environments in starch systems typically reduce peak viscosity and breakdown [37,38]. According to these authors, alkaline treatment weakens the structure of starch granules, facilitating their disruption during thermal processing [39]. In our study, although the trough viscosity increased and the breakdown decreased, suggesting greater paste stability, the peak viscosity remained unaffected, indicating a differential behavior compared to traditional starch systems.

In this context, Xiao et al. [40] observed that in rice flour, peak viscosity, trough viscosity, and final viscosity increased with sodium bicarbonate concentrations ≤ 0.1% but decreased when the concentration was increased to between 0.1% and 0.5%. This behavior suggests that low concentrations of bicarbonate may stabilize the starch structure, whereas higher concentrations promote its degradation.

On the other hand, the removal of sodium bicarbonate in the FB formulation showed no significant differences compared to the control formulation (RF), nor with respect to FA. This suggests that the additional removal of sodium bicarbonate had no clear impact on these parameters, reinforcing the idea that monocalcium phosphate is the key component modulating starch paste stability in this system.

It is worth noting that the specific characteristics of the gluten-free flour used may influence its interaction with other formulation components, potentially explaining the observed differences compared to previous studies on traditional flours. Several studies have demonstrated that the incorporation of psyllium significantly increases viscosity throughout the RVA curve [41,42,43]. Specifically, Belorio et al. [41] reported that psyllium enhances viscosity during gelatinization, particularly at low concentrations (2–5%), but its effect is more pronounced in promoting setback (retrogradation) rather than altering peak viscosity. This behavior suggests that psyllium stabilizes the starch paste structure after gelatinization, limiting granule swelling and thus preventing drastic changes in peak viscosity.

Additionally, Chaisawang et al. [44] observed that xanthan gum can delay gelatinization and reduce peak viscosity in tapioca starch systems due to electrostatic repulsion between negatively charged groups. This interaction may also contribute to the stability of the viscosity profile in gluten-free formulations containing both hydrocolloids.

The removal of monoglyceride in the FC resulted in significant differences in gelatinization temperature compared to the other formulations. This is consistent with previous studies demonstrating that monoglycerides can establish specific interactions with starch granules, forming amylose–lipid complexes that modify the gelatinization process [20,21,22].

Guven et al. [20] incorporated 1% monoglyceride, composed of monopalmitin and monostearin, into wheat flour and observed that its addition delayed pasting, requiring higher temperatures and longer times to reach peak viscosity, although the peak value itself was not affected. Similarly, Zhou et al. [45] reported that stearic acid in rice starch markedly reduced peak viscosity and breakdown, while increasing the time to peak viscosity, whereas linoleic acid exerted a much more limited effect. In agreement with these findings, Kaur et al. [21] observed that the incorporation of fatty acids into rice flour increased pasting temperature and paste viscosity, an effect attributed to the formation of amylose–lipid complexes that limit water transport into starch granules. Along the same lines, Lai et al. [22] reported that the addition of distilled glyceryl monostearate to rice flour increased swelling temperature by 18 °C and enhanced paste viscosity. Taken together, these results suggest that the monoglyceride employed in this study, mainly composed of long-chain saturated fatty acids such as stearic and palmitic acid, may have contributed to the increase in gelatinization temperature.

#### 3.1.2. Physicochemical Analysis of Bread

The removal of additives had a varied impact on the physicochemical characteristics of gluten-free breads at day 1 of storage (Table 3).

The bake loss values (Table 3) did not show significant differences (*p* > 0.05) among the formulations, ranging from 8% (RF) to 10% (FA). This suggests that the removal of additives did not affect the water loss during baking, maintaining the process efficiency across all treatments.

**Figure 1:**
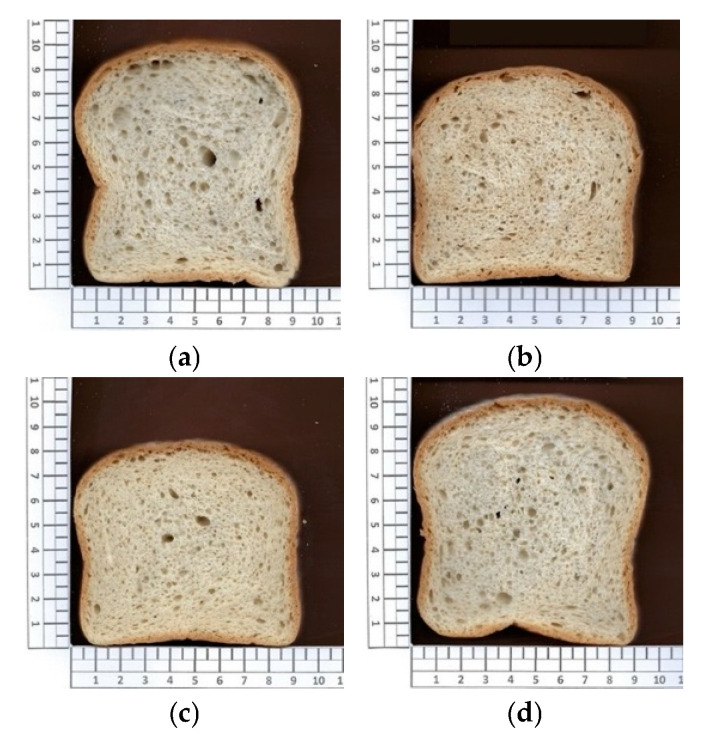
Slice images of gluten-free bread samples for the four formulations: (**a**) RF: Reference Formulation; (**b**) FA: Removal of monocalcium phosphate; (**c**) FB: Removal of monocalcium phosphate and sodium bicarbonate; (**d**) FC: Removal of monocalcium phosphate, sodium bicarbonate, and mono- and diglycerides of fatty acids.

Specific volume is a critical quality parameter in bread, as it directly influences consumer acceptance [46]. In this study, the progressive removal of additives had a significant impact on the specific volume of the different formulations (Figure 1).

Formulation FB (without monocalcium phosphate and sodium bicarbonate) exhibited the lowest specific volume (2.8 cm^3^/g), which was significantly lower than that of the reference formulation (RF). This result reinforces the key role of these leavening agents in gas generation during baking [47]. The visual differences in loaf expansion are evident in Figure 1c, where the reduced volume of FB is clearly observable.

These results suggest that monocalcium phosphate and sodium bicarbonate, acting as leavening agents, positively influence bread specific volume. However, when analyzing the effect of emulsifiers, an opposite trend was observed. The removal of mono- and diglycerides in the FC resulted in a higher specific volume (3.3 cm^3^/g), indicating that, in the absence of chemical leavening agents, mono- and diglycerides did not contribute to volumetric development in this gluten-free system.

Similar behavior was reported by Yeşil et al. [26], who observed a reduction in specific volume when monoglycerides were added to a gluten-free formulation based on rice flour and corn starch with sunflower oil as the fat source. Likewise, Hellsten [48] reported increased specific volume after removing mono- and diglycerides in a system containing rice flour, corn starch, potato starch, and rapeseed oil. These studies suggest that, in certain gluten-free formulations, monoglycerides may exert a restrictive effect on gas expansion, potentially related to changes in dough viscosity or gas cell stabilization.

In contrast, Nunes et al. [49] reported an increase in specific volume following the addition of distilled monoglycerides in a gluten-free formulation containing rice flour, potato starch, and margarine, highlighting that the technological functionality of emulsifiers strongly depends on formulation variables such as the lipid source and matrix composition.

In wheat-based systems, where gluten forms a continuous elastic network that promotes gas retention, the role of monoglycerides appears to be even more formulation-dependent. Gómez et al. [50] reported that their effect on bread volume was optimal at intermediate fermentation times but decreased with prolonged fermentation, likely due to alterations in gluten network integrity. Similarly, Guven et al. [20] showed that monoglycerides did not significantly affect bread volume in wheat–psyllium systems, where the high water-holding capacity of psyllium was the dominant factor negatively influencing specific volume.

Overall, these findings indicate that the impact of mono- and diglycerides on bread volume is highly dependent on the formulation and structural framework of the dough, which explains the contrasting effects reported in the literature and is consistent with the behavior observed in the present study.

Regarding moisture content, breads from formulation FB exhibited slightly lower values compared to the other formulations, although differences were not consistently significant. Similarly, water activity (aw) remained high in all samples (above 0.98) and did not differ significantly among formulations. These results indicate that water availability was not markedly affected by additive removal and therefore did not play a determining role in the differences observed in crumb structure and specific volume.

Digital image analysis was used to evaluate the effect of progressive additive removal on crumb cell structure. Overall, no significant differences were observed in the total number of cells or in the number of small cells among formulations, suggesting that all breads developed a comparable alveolar network in terms of cell density. In contrast, parameters related to gas cell size and porosity were strongly affected by formulation, indicating differences in gas expansion and stabilization during baking.

In formulation FA (without monocalcium phosphate), a more open and aerated crumb structure was obtained, as reflected by a significantly higher void fraction (30.4). This formulation also exhibited the highest number of large cells (68), although differences with RF and FC were not statistically significant. These results suggest that the removal of monocalcium phosphate, in the presence of sodium bicarbonate and mono- and diglycerides, promoted more effective gas cell expansion during baking, possibly due to a more gradual gas release. In agreement with this interpretation, Blanco et al. [51] reported that the presence of monosodium phosphate in gluten-free bread was associated with larger alveolus size and higher loaf volume, an effect attributed to its interaction with structuring agents such as HPMC, highlighting the role of phosphates in modulating gas cell development.

Conversely, formulation FB (without monocalcium phosphate and sodium bicarbonate, but with mono- and diglycerides) exhibited a more compact crumb structure, characterized by the smallest mean cell area (0.64 ± 0.03 mm^2^) and the lowest number of large cells (47), significantly lower than those observed in RF and FA. The void fraction was also the lowest (27.0), although it only differed significantly from FA. This combination of structural parameters indicates a reduced capacity for gas expansion, consistent with the lower specific volume observed in this formulation. The simultaneous absence of both leavening agents likely limited CO_2_ generation during baking, resulting in restricted bubble growth and a denser crumb. In this context, the presence of mono- and diglycerides may have further contributed to limiting gas cell expansion, consistent with the restrictive effect on specific volume discussed above and with the findings of Yeşil et al. [26], who reported reduced loaf volume in gluten-free bread when monoglycerides were used alone. Moreover, Ndjang et al. [52] observed that sodium bicarbonate exerts a positive effect on the specific volume of gluten-free bread, supporting the limited volumetric development observed in its absence.

Finally, formulation FC (without monocalcium phosphate, sodium bicarbonate, and mono- and diglycerides) did not differ significantly from RF in terms of crumb structure parameters, despite the complete removal of additives. Although it did not exhibit the highest void fraction, FC showed the greatest overall specific volume. This behavior suggests that, in the absence of emulsifiers, gas expansion during baking was sufficient to increase loaf volume without promoting excessive gas cell coalescence. However, it should be noted that image-based porosity assessment may underestimate cell area in foam-like structures with low contrast between gas cells and cell walls. As reported by Mezaize et al. [53], this limitation can affect the accuracy of crumb structure quantification in highly aerated systems.

The color of the crumb and crust of the bread was evaluated on day 1. L*, a*, and b* data were collected for all breads, and the total color difference (ΔE1*) was calculated for FA, FB, and FC compared to RF. The effect of additive removal on the color parameters of gluten-free bread crumb and crust is presented in Table 4.

Overall, the progressive removal of additives resulted in measurable changes in bread color. Although total color differences in the crumb were moderate and statistically comparable among the reduced-additive formulations, all showed perceptible deviations relative to the reference bread. In fact, ΔE_1_ values for crumb ranged between 3.1 and 3.9, exceeding the threshold commonly associated with clearly perceptible color differences (ΔE* > 3) [54]. Among the reduced-additive formulations, FA exhibited the highest ΔE_1_* value (3.9), whereas FC showed the lowest deviation from the reference formulation, indicating that the complete removal of additives resulted in the smallest color change relative to RF.

When individual color coordinates were considered, differences among formulations became more evident. In the crumb, formulation FB exhibited higher lightness (L*) values, suggesting a paler appearance, whereas formulation FA, which contained sodium bicarbonate, showed significantly higher a* values (*p* < 0.05) together with higher b* values, indicating a shift toward more reddish and yellowish tones. In contrast, formulation FC did not show significant differences in L*, a*, or b* values compared to the reference formulation, indicating a crumb color closely comparable to that of RF.

Previous studies have reported that increasing the level of sodium bicarbonate leads to higher b* values, reflecting a more yellowish appearance. In particular, Xiao et al. [40], working with rice noodles, observed an increase in b* values with increasing sodium bicarbonate addition. In the present study, however, the formulation containing sodium bicarbonate but lacking monocalcium phosphate (FA) exhibited increases in both a* and b* values, whereas formulation FB, in which both leavening agents were removed, showed an increase in L* without a corresponding decrease in b* values, which remained comparable to those of the reference formulation despite the removal of sodium bicarbonate. These observations suggest that, under the conditions evaluated, variations in crumb color may be associated with differences in loaf expansion and crumb structure rather than with direct chemical browning effects.

In contrast to the crumb, color differences in the crust were more pronounced. ΔE_1_* values for the crust ranged from 7 to 10, indicating clearly perceptible visual differences among formulations. These results show that changes in formulation led to more evident modifications in crust appearance than in crumb color under the conditions evaluated.

When individual color coordinates were examined, formulation-dependent trends became apparent. Formulation FC exhibited significantly higher L* values than the reference formulation (*p* < 0.05), indicating a lighter crust, whereas formulation FB showed the highest a* and b* values, resulting in a more reddish and golden surface. Overall, these observations indicate a greater sensitivity of crust color to formulation changes compared to crumb color in the present study.

The removal of additives had a significant impact on the texture of gluten-free breads at day 1 of storage, affecting both crumb and crust characteristics (Table 5).

Texture is one of the main attributes determining the perception of freshness in bakery products, particularly in bread, where crumb firmness and elasticity directly influence consumer acceptance [55]. Among the different textural attributes, hardness is the one that shows the highest correlation between sensory and instrumental measurements [56].

Formulation FB, which did not contain monocalcium phosphate or sodium bicarbonate, exhibited the highest crumb hardness (37.9), followed by FA (30.1). In contrast, the RF, which included all additives, showed the lowest hardness (18.0), indicating a significantly softer crumb. These results suggest that additives, particularly leavening agents, play a key role in contributing to the initial softness of gluten-free bread.

Previous studies on flatbread have demonstrated that the exclusive use of sodium bicarbonate results in a firmer texture, while its combination with yeast reduces bread hardness compared to yeast alone [57,58]. Zolfaghari et al. [58] also reported that bicarbonate may enhance α-amylase activity, thereby improving fermentation—by increasing sugar availability—and reducing bread hardness by preventing excessive starch gelatinization.

Similarly, Xiao et al. [40] observed that the addition of sodium bicarbonate affects the hardness of rice-based products by modifying starch structure. At low concentrations (0–0.1%), hardness increased due to recrystallization promoted after disruption of amorphous regions, whereas at higher concentrations (0.1–0.5%), hardness decreased because of reduced starch–water interactions and altered microcrystalline organization.

In addition, Blanco et al. [51] found that the presence of monosodium phosphate in gluten-free bread improved both hardness and specific volume, as phosphate forms hydrogen bonds with the HPMC network, promoting CO_2_ retention during fermentation and enhancing the final texture.

Finally, formulation FC, which lacked both leavening agents and mono- and diglycerides, presented intermediate hardness, although significantly lower than that of FB and FA. This effect could be attributed to the higher specific volume observed in FC, as several studies have reported that increased specific volume is associated with decreased bread hardness [27,32,46,59].

Cohesiveness is defined as the degree to which the crumb maintains its structural integrity during compression or mastication [56]. For this parameter, values ranged between 0.62 and 0.68. Formulation FB exhibited the lowest cohesiveness (0.62), which was significantly lower than that of FA (0.68), indicating a more fragile internal structure and a lower ability to resist deformation. In contrast, formulations RF (0.66) and FC (0.66) showed intermediate values, with no significant differences between them or in comparison with FA and FB.

These results suggest that, although cohesiveness was not strongly affected by the individual removal of additives, the simultaneous removal of monocalcium phosphate and sodium bicarbonate (FB) weakened the structural network of bread. Conversely, formulation FC, in which all additives were removed, exhibited cohesiveness comparable to the reference formulation (RF).

Regarding springiness, all samples exhibited high values (>0.939). High crumb elasticity is desirable, as it is associated with greater freshness perception and a more elastic bread texture—attributes positively valued by consumers [34]. Formulation FC showed the highest value (0.984), significantly higher than FB, although no significant differences were observed compared to RF and FA. These results suggest that the presence of mono- and diglycerides of fatty acids may negatively affect crumb elasticity, whereas the addition of leavening agents contributes to its improvement.

For chewiness, a trend like hardness was observed. Formulations FB and FA recorded the highest values (22.0 N and 19.7 N, respectively), while RF and FC exhibited significantly lower chewiness, consistent with their softer and more elastic crumb structure.

Similarly, gumminess showed the same tendency, with FB and FA presenting significantly higher values than RF and FC.

The crust puncture force ranged between 1.15 and 1.58 N. Formulation FA (without monocalcium phosphate) showed the highest value (1.58 N), significantly higher than RF, indicating a more rigid and resistant crust. It was followed by FB (1.40 N), which lacked leavening agents but contained monoglycerides, whereas RF (1.21 N) and especially FC (1.15 N) exhibited lower values, the latter showing the softest crust.

These results suggest that formulations without leavening agents but containing monoglycerides (FA and FB) developed a harder crust, possibly related to their lower specific volume. In contrast, the simultaneous removal of all additives (FC) resulted in a thinner and more fragile crust, probably associated with its higher specific volume and lower surface rigidity.

This behavior could be attributed to the effect of sodium bicarbonate, which, when acting without neutralizing acid, increases the system’s pH and may contribute to higher bread hardness [57].

### 3.2. Bread Staling During Storage

Bread staling is a complex phenomenon involving physical and chemical changes that affect the texture, structure, and visual attributes of the product over time. In gluten-free breads, these changes may be even more pronounced due to the absence of gluten and the specific role of functional additives. This section presents the evolution of texture and color parameters of breads from different formulations during 22 days of storage, aiming to assess the impact of additive removal on bread quality preservation.

#### 3.2.1. Texture

The delay in staling in bakery products has been the focus of considerable industrial and academic interest, although the mechanisms underlying this process are not yet fully understood [60]. This phenomenon is typically characterized by a progressive increase in crumb hardness during storage, accompanied by a reduction in cohesiveness, springiness, and structural resilience [61]. In addition to its sensory impact, bread staling decreases consumer acceptance and leads to significant economic losses in the bakery sector [62].

**Figure 2:**
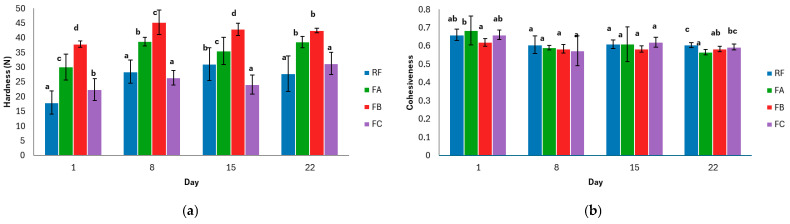

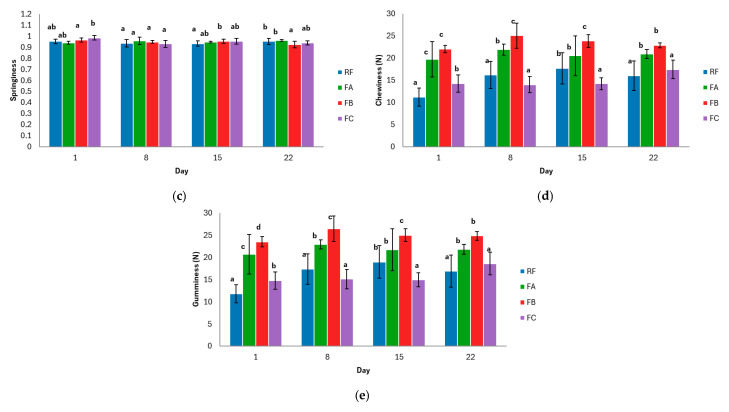
(**a**) Hardness evolution during storage; (**b**) cohesiveness evolution during storage; (**c**) springiness evolution during storage; (**d**) chewiness evolution during storage; (**e**) gumminess evolution during storage. Data are presented as mean values ± standard deviation. Two independent dough batches were prepared per formulation, and four slices per batch were analyzed for texture profile analysis. Different letters indicate significant differences (*p* < 0.05) among formulations at the same storage time. RF: Reference Formulation. FA: Removal of monocalcium phosphate. FB: Removal of mono-calcium phosphate and sodium bicarbonate. FC: Removal of monocalcium phosphate, sodium bicarbonate, and mono- and diglycerides of fatty acids.

Figure 2 shows the texture profiles (hardness, cohesiveness, springiness, chewiness, and gumminess) obtained for each formulation during storage (days 1, 8, 15, and 22).

The evolution of crumb hardness (Figure 2a) showed a progressive increase in all formulations throughout storage, confirming the effect of staling. However, the magnitude of this hardening clearly depended on the composition of each formulation. For RF, FA, and FB, the trend observed was consistent with the initial values: FB remained the formulation with the highest hardness, followed by FA, and finally RF as the softest.

In contrast, the behavior of formulation FC differed from what would be expected. Although it exhibited higher hardness than RF on day 1, this difference was no longer significant on days 8 and 22, and on day 15, FC even showed lower hardness values than RF. This result suggests that the complete removal of additives did not necessarily lead to a faster hardening rate, and that the higher specific volume of FC may have contributed to delaying firmness development during storage.

Cohesiveness reflects the degree to which the crumb can remain intact after deformation and is associated with the internal integrity of the bread matrix during mastication [56]. All formulations showed a slight decrease in this parameter throughout storage, which is consistent with the expected behavior during the staling process, in which starch retrogradation and moisture migration progressively weaken the structural capacity of the crumb [63].

Although cohesiveness decreased gradually in all samples, RF maintained the highest structural integrity at the end of storage, indicating greater resistance to mechanical breakdown. In contrast, FA exhibited the greatest relative loss of cohesiveness, suggesting reduced stability of its internal network over time. FB—lacking leavening agents—showed the lowest value on day 1 but evolved to an intermediate level between FA and FC by the end of storage. Finally, formulation FC, despite containing no additives, maintained cohesiveness values comparable to RF, suggesting that its higher initial specific volume may have helped preserve cohesion during storage.

Over the 22-day storage period, the springiness of the gluten-free bread crumb remained relatively high, although the magnitude of the changes depended on the formulation. Initially, all formulations exhibited similar springiness values, with only minor differences. As storage progressed, slight decreases were observed across all samples.

Formulation FB, in which both monocalcium phosphate and sodium bicarbonate were removed, showed the greatest decline in springiness over time, suggesting that these leavening agents contribute to maintaining crumb elasticity during storage. In contrast, formulation FA, where only monocalcium phosphate was omitted, maintained high springiness levels comparable to those of the reference formulation (RF), indicating that the absence of monocalcium phosphate alone did not negatively affect this parameter.

Formulation FC, which lacked monocalcium phosphate, sodium bicarbonate, and mono- and diglycerides of fatty acids, displayed intermediate behavior, showing no significant differences from the other formulations at the final measurement.

Overall, the results indicate that sodium bicarbonate plays a key role in preserving crumb elasticity during storage, as its absence (FB) was associated with the greatest loss of springiness. In contrast, the removal of monocalcium phosphate had only a limited impact under the conditions evaluated, since FA maintained values like those of the control formulation. Regarding mono- and diglycerides, their removal appeared to slightly enhance elasticity (FC), placing this formulation at intermediate levels by the end of storage.

Chewiness and gumminess increased in all formulations throughout storage, following a pattern consistent with the evolution of hardness. The magnitude of these changes, however, depended strongly on the additives removed.

Formulation FB—lacking both monocalcium phosphate and sodium bicarbonate—consistently exhibited the highest chewiness and gumminess values at all storage times. This behavior aligns with its denser crumb structure and higher hardness, suggesting that the absence of both leavening agents promotes a more compact and resistant matrix. Formulation FA (without monocalcium phosphate) also showed elevated values, although to a lesser extent than FB.

In contrast, RF (the control formulation containing all additives) and FC (without any additives) maintained the lowest chewiness and gumminess values throughout storage. While RF showed a moderate increase over time, FC remained relatively stable. FC did not exhibit exacerbated chewiness or gumminess, likely due to its higher initial specific volume.

These observations differ from those reported by Güven et al. [20], who found no significant changes in chewiness when mono- and diglycerides were added to gluten-free breads; however, in their study, the inclusion of these emulsifiers did not reduce specific volume, which likely mitigated their impact on textural parameters.

Overall, the results indicate that the removal of gas-releasing agents—particularly when both monocalcium phosphate and sodium bicarbonate are omitted—substantially increases chewiness and gumminess due to the formation of a denser and more compact crumb. Conversely, the presence of all additives (RF) helped limit the development of these staling-associated attributes.

**Figure 3:**
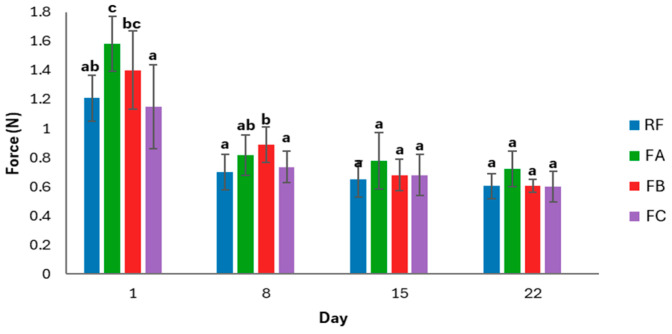
Puncture force evolution during storage. Data are presented as mean values ± standard deviation. Two independent dough batches were prepared per formulation, and four slices per batch were analyzed for crust puncture tests. Different letters indicate significant differences (*p* < 0.05) among formulations at the same storage time. RF: Reference formulation. FA: Removal of monocalcium phosphate. FB: Removal of monocalcium phosphate and sodium bicarbonate. FC: Removal of monocalcium phosphate, sodium bicarbonate, and mono- and diglycerides of fatty acids.

Figure 3 presents the evolution of crust puncture force in gluten-free breads from different formulations over 22 days of storage. This parameter reflects changes in crust firmness during staling and highlights how the removal of specific additives influenced the mechanical resistance of the crust throughout the storage period.

Crust puncture force decreased progressively in all formulations during storage, indicating a general weakening of crust structure over time. This behavior is consistent with the moisture migration from the crumb to the crust described by Besbes et al. [64], who reported that the crust gradually absorbs part of the water released from the crumb during staling, leading to a softer and less firm crust. On day 1, formulation FA (without monocalcium phosphate) exhibited the highest puncture force, significantly higher than RF and FC. FB (without monocalcium phosphate and sodium bicarbonate) also showed relatively high initial values, whereas FC (without any additives) presented the softest crust.

During the first week, all samples experienced a marked decline in puncture force. On day 8, differences were still evident: FB maintained a firmer crust than RF and FC, while FA showed intermediate values. From day 15 onward, no significant differences were detected among formulations, and by day 22, all samples exhibited similarly low crust firmness.

These results suggest that formulations with reduced specific volume (FA and FB) developed a thicker and firmer crust at the beginning of storage, whereas FC, with the highest specific volume, formed a softer and more fragile crust. However, this initial effect diminished over time, and the progressive softening of the crust followed a similar pattern across all formulations.

Overall, the removal of additives had only a short-term influence on crust mechanical properties, and long-term crust deterioration appeared to be primarily driven by moisture migration during storage rather than by formulation differences.

#### 3.2.2. Color

Color changes are important quality indicators in gluten-free breads, as they can influence consumer perception of freshness and acceptability during storage. In this section, the evolution of crust and crumb color is analyzed through CIELab parameters (ΔL*, Δa*, Δb*, and ΔE_2_*), calculated by comparing each formulation with its own initial color values on day 1. This approach allows us to assess the magnitude and direction of color changes over time within each formulation.

**Figure 4:**
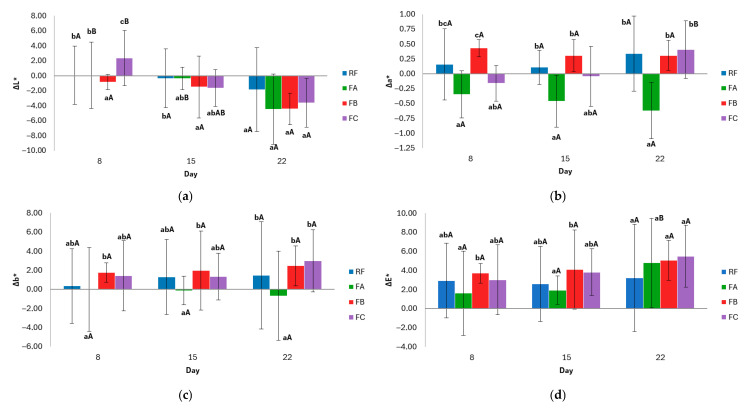
Evolution of crumb color parameters during storage: (**a**) ΔL* (lightness), (**b**) Δa* (red–green coordinate), (**c**) Δb* (yellow–blue coordinate), and (**d**) ∆E* (total color difference). Values mean ± standard deviation. Two independent dough batches were prepared per formulation, and three slices per batch were analyzed for color measurements. Different lowercase letters indicate significant differences (*p* < 0.05) between formulations within the same day; different uppercase letters indicate significant differences (*p* < 0.05) within the same formulation over time. RF: Reference Formulation. FA: Removal of monocalcium phosphate. FB: Removal of monocalcium phosphate and sodium bicarbonate. FC: Removal of monocalcium phosphate, sodium bicarbonate, and mono- and diglycerides of fatty acids.

Overall, formulation-dependent differences were observed in the evolution of crumb color during storage, although not all parameters were equally affected. The evolution of crumb color parameters during storage is shown in Figure 4.

The ΔL* parameter represents changes in crumb lightness. All formulations generally exhibited a decrease in lightness, indicating darkening relative to day 1. On day 8, the FB formulation showed a significantly greater reduction in lightness than the RF and FA formulations, suggesting greater susceptibility to browning at an earlier storage stage. In contrast, the FC exhibited a unique pattern with an initial increase in lightness on day 8, followed by a progressive darkening. Over time, the formulations stabilized, with no significant differences observed between days within the same formulation (*p* > 0.05).

The Δa* parameter, which reflects the red–green chromatic component of the crumb, showed formulation-dependent trends. The FB formulation exhibited the highest positive Δa∗ values, indicating a redder crumb while maintaining its chromatic stability over time. Conversely, the FA formulation presented consistently negative Δa* values (greener), also with temporal stability. The RF showed low positive and stable values. Notably, the FC exhibited a significant change (*p* < 0.05) in a* component over time, shifting from a greenish to a more neutral and slightly reddish hue by day 22.

The Δb* parameter indicates the variation in the yellow–blue component of crumb color. A general trend towards positive values was observed in the FB and FCs throughout storage, particularly on day 22. The RF remained near baseline, with low and stable Δb* values, indicating high chromatic stability. In contrast, the FA formulation showed a progressive decrease in Δb* values over time, reaching negative values by day 22, which suggests a gradual loss of yellow intensity.

Concerning the total color difference (ΔE_2_*) of the crumb during storage, all formulations exhibited perceptible changes (ΔE_2_* > 3.0) throughout the evaluated period. From the outset of storage, the FB and FCs exhibited a noticeable color change, whereas the RF exhibited a value close to the threshold, indicating the onset of visual alteration. Conversely, FA exhibited superior color stability in the initial stages. As storage progressed, all formulations experienced an increase in ΔE*, which was more pronounced in FA, FB, and FC by day 22. Statistical analysis indicates that there was a significant difference between the last day and previous days for FA (*p* < 0.05), while there were no significant variations between days for RF, FB, and FC, but there were significant variations between formulations at specific times. This suggests that the type and combination of additives influence color evolution. RF, which contains monocalcium phosphate and sodium bicarbonate, exhibited a more stable evolution, while FA, which contains only bicarbonate, exhibited a later but more pronounced change. Meanwhile, FB and FC, which do not contain leavening agents, exhibited an earlier and more sustained visual alteration.

**Figure 5:**
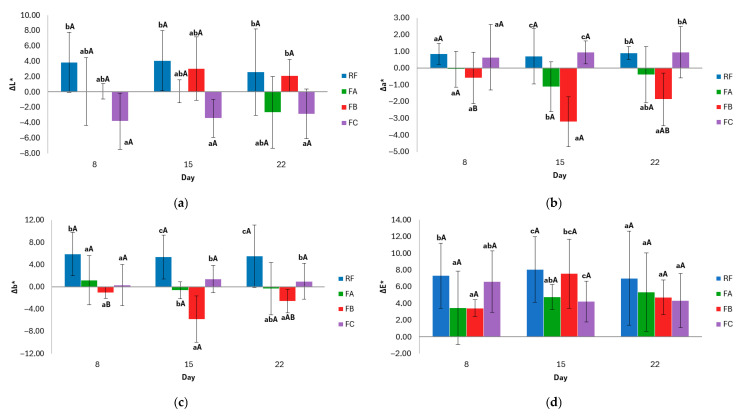
Evolution of crust color parameters during storage. (**a**) ΔL* (lightness), (**b**) Δa* (red–green coordinate), (**c**) Δb* (yellow–blue coordinate), and (**d**) ∆E* (total color difference). Values mean ± standard deviation. Two independent dough batches were prepared per formulation, and three slices per batch were analyzed for color measurements. Different lowercase letters indicate significant differences (*p* < 0.05) between formulations within the same day; different uppercase letters indicate significant differences (*p* < 0.05) within the same formulation over time. RF: Reference Formulation. FA: Removal of monocalcium phosphate. FB: Removal of monocalcium phosphate and sodium bicarbonate. FC: Removal of monocalcium phosphate, sodium bicarbonate, and mono- and diglycerides of fatty acids.

The evolution of crust color during storage was evaluated using CIELab parameters (ΔL*, Δa*, Δb*, and ΔE_2_*), calculated relative to the initial color values on day 1 for each formulation. Given the greater exposure of the crust to oxygen and thermal effects during baking, more pronounced and dynamic color changes were expected compared to the crumb. The evolution of crust color parameters during storage is shown in Figure 5.

Regarding ΔL*, formulation RF exhibited a sustained increase in lightness during storage, indicating a progressively lighter crust over time. Formulations FA and FB showed moderate increases in lightness, whereas formulation FC exhibited negative ΔL* values throughout storage, indicating progressive darkening of the crust. This trend suggests that, in the absence of additives, crust darkening may occur earlier or more steadily during storage.

For the red–green coordinate (Δa*), formulations RF and FC exhibited positive and relatively stable values, indicating a slight intensification of reddish tones. In contrast, formulation FA showed moderately negative Δa* values, while formulation FB exhibited a significant decrease in the red component on day 15 (*p* < 0.05), suggesting a reduction in warm color tones at intermediate storage stages.

Changes in the yellow–blue coordinate (Δb*) also depended on formulation. RF consistently exhibited positive and relatively high Δb* values, indicating an intensification of yellowish tones in the crust. Formulations FA and FC showed low or slightly positive Δb* values, whereas formulation FB exhibited negative Δb* values throughout storage, indicating a progressive loss of yellowness. Significant differences between formulations (*p* < 0.05) suggest that FB tended to develop cooler color tones over time.

Considering the total color difference (ΔE_2_*), all formulations reached values perceptible to the human eye (ΔE_2_* > 3.0) at some point during storage. The reference formulation (RF) exhibited the highest ΔE_2_* values, indicating a greater overall color change in the crust during storage. Formulation FB, containing mono- and diglycerides but lacking leavening agents, also showed marked color variation. In contrast, formulation FA exhibited a more gradual evolution, with a significant increase in ΔE_2_* observed only between the final storage day and the preceding one (*p* < 0.05). Formulation FC, without additives, showed a slower but consistent progression of color change from the initial stages.

Overall, these results indicate that crust color is more sensitive to storage time and formulation than crumb color. The magnitude and timing of crust color changes depended on the type and combination of additives present, confirming that additive removal modulates the dynamics of color evolution during storage rather than completely preventing visual changes.

## 4. Conclusions

The progressive removal of monocalcium phosphate, sodium bicarbonate, and mono- and diglycerides significantly affected the initial quality of gluten-free bread, primarily influencing specific volume and crumb structure. While sodium bicarbonate and monocalcium phosphate proved essential for gas generation and expansion during baking—with their absence reducing loaf volume and increasing initial hardness, particularly in formulation FB—the complete removal of additives (FC) not only preserved initial quality but also produced the highest specific volume and a crumb structure comparable to the reference formulation (RF). This demonstrates that mono- and diglycerides are not indispensable for achieving adequate expansion in gluten-free systems.

During storage, all formulations exhibited a typical staling pattern, characterized by increased hardness, decreased cohesiveness, and springiness. However, formulation FC did not show a faster staling rate than RF, suggesting that its greater initial expansion contributed to delaying firmness development.

Overall, these results confirm that leavening agents are critical for the initial structure of gluten-free bread, but they also reveal that the removal of mono- and diglycerides is a viable strategy for developing clean-label formulations without compromising textural evolution during storage. This approach not only addresses the growing demand for more natural products but also opens new possibilities for optimizing gluten-free formulations with fewer synthetic additives, aligning with current trends in the food industry.

The findings of this study have direct implications for the gluten-free baking industry, where there is increasing demand for clean-label products. The removal of mono- and diglycerides—without compromising gluten-free bread quality—opens opportunities to formulate products with fewer additives, aligning with consumer preferences for natural and minimally processed foods. Optimizing leavening agents (e.g., sodium bicarbonate and monocalcium phosphate) is critical to maintain volume and texture, guiding the development of new gluten-free bread formulations with improved sensory and structural properties. This approach could also be extended to other gluten-free baked goods, such as cakes or muffins, where texture and volume are key quality attributes.

Future research should explore the use of sourdough fermentation as a potential natural strategy to reduce the reliance on synthetic preservatives in gluten-free bread. Since sourdough fermentation leads to the production of organic acids (e.g., lactic and acetic acids), which may contribute to enhanced microbial stability and extended shelf life, further studies are required to evaluate their effectiveness specifically in gluten-free systems. In this context, the impact of sourdough on the textural, sensory, and antimicrobial properties of gluten-free bread warrants particular attention, as its application could support the development of cleaner-label formulations while maintaining overall product quality.

In addition, the potential synergistic effects of combining sourdough fermentation with natural emulsifiers or structuring agents (e.g., lecithin or hydrocolloids) should be investigated as a strategy to further reduce or eliminate synthetic additives. Likewise, the role of starch modifications (such as pregelatinized or resistant starches) and their interactions with sourdough-derived components may provide valuable insights into improving both quality attributes and shelf-life stability in clean-label gluten-free bread.

Finally, long-term storage studies, including comprehensive microbial stability assessments and consumer acceptance evaluations, will be essential to fully determine the technological feasibility and commercial potential of these formulations.

## Figures and Tables

**Table 1:** Composition of the formulations in the study (g/100g gluten-free baking mix).

	Formulation			
Ingredient	RF	FA	FB	FC
Gluten-free bread mix	100	100	100	100
Monocalcium phosphate	1	0	0	0
Sodium bicarbonate	0.7	0.7	0	0
Mono- and diglycerides of fatty acids	0.3	0.3	0.3	0

RF: Reference Formulation. FA: Removal of monocalcium phosphate. FB: Removal of monocalcium phosphate and sodium bicarbonate. FC: Removal of monocalcium phosphate, sodium bicarbonate, and mono- and diglycerides of fatty acids.

**Table 2:** Mean values ± standard deviations of RVA pasting properties of the mix formulation.

Formulation	RF	FA	FB	FC
Pasting Temperature (°C)	73.50 ± 1.13 ^b^	74 ± 2 ^b^	74 ± 3 ^b^	50.19 ± 0.06 ^a^
Peak Time (min)	4.84 ± 0.05 ^a^	5.1 ± 0.5 ^a^	5.3 ± 0.3 ^a^	4.71 ± 0.05 ^a^
Peak Viscosity (cP)	4186 ± 416 ^a^	4025 ± 159 ^a^	3907 ± 7 ^a^	4153 ± 15 ^a^
Trough (cP)	2219 ± 130 ^a^	2733 ± 143 ^b^	2412 ± 312 ^ab^	2562 ± 31 ^ab^
Breakdown (cP)	1967 ± 286 ^b^	1292 ± 16 ^a^	1496 ± 305 ^ab^	1592 ± 46 ^ab^
Final Viscosity (cP)	3026 ± 302 ^a^	3659 ± 507 ^a^	3384 ± 118 ^a^	3408 ± 21 ^a^
Setback (cP)	807 ± 432 ^a^	926 ± 364 ^a^	972 ± 430 ^a^	846 ± 10 ^a^

Data are presented as mean values ± standard deviation. Two independent dough batches were prepared per formulation. Different superscript letters within the same column indicate homogeneous groups according to ANOVA (*p* < 0.05). RF: Reference Formulation. FA: Removal of monocalcium phosphate. FB: Removal of monocalcium phosphate and sodium bicarbonate. FC: Removal of monocalcium phosphate, sodium bicarbonate, and mono- and diglycerides of fatty acids.

**Table 3:** Mean values ± standard deviations of Physicochemical properties of gluten-free breads with different additive removal treatments (day 1 of storage).

Formulation	RF	FA	FB	FC
Bake Loss (%)	8 ± 3 ^a^	10 ± 2 ^a^	9.2 ± 1.9 ^a^	9 ± 3 ^a^
Specific volume (cm^3^/g)	3.0 ± 0.2 ^b^	2.9 ± 0.3 ^ab^	2.8 ± 0.2 ^a^	3.3 ± 0.4 ^c^
Bread CM (%)	44.8 ± 0.4 ^ab^	45.2 ± 0.3 ^b^	43.4 ± 1.8 ^a^	45.0 ± 1.5 ^ab^
Bread a_w_	0.984 ± 0.005 ^a^	0.986 ± 0.007 ^a^	0.988 ± 0.003 ^a^	0.986 ± 0.004 ^a^
Total Cells	969 ± 102 ^a^	1058 ± 103 ^b^	1048 ± 46 ^ab^	983 ± 57 ^ab^
Area cell media (mm^2^)	0.73 ± 0.07 ^b^	0.73 ± 0.09 ^b^	0.64 ± 0.03 ^a^	0.71 ± 0.06 ^ab^
Small cells	907 ± 107 ^a^	991 ± 116 ^ab^	1002 ± 53 ^b^	928 ± 59 ^ab^
Large cells	62 ± 12 ^b^	68 ± 14 ^b^	47 ± 8 ^a^	55 ± 12 ^ab^
Void Fraction (%)	28 ± 2 ^a^	30.4 ± 1.8 ^b^	27.0 ± 0.5 ^a^	28 ± 2 ^a^

Data are presented as mean values ± standard deviation. Two independent dough batches were prepared per formulation, with ten loaves obtained from each batch. Baking loss and specific volume were determined in all loaves (*n* = 20). Moisture content and water activity were analyzed in four slices (two per batch). Digital image analysis (total number of cells, mean cell area, small and large cells, and void fraction) was performed on six slices (three per batch). Different superscript letters indicate significant differences (ANOVA, *p* < 0.05). RF: Reference Formulation. FA: Removal of monocalcium phosphate. FB: Removal of monocalcium phosphate and sodium bicarbonate. FC: Removal of monocalcium phosphate, sodium bicarbonate, and mono- and diglycerides of fatty acids.

**Table 4:** Mean values ± standard deviations of color values (crumb and crust) of gluten-free breads with different additive removal treatments at day 1 of storage.

Formulation		RF	FA	FB	FC
Crumb	L*	70 ± 3 ^ab^	69 ± 2 ^a^	72.8 ± 1.2 ^b^	70 ± 4 ^ab^
	a*	−0.5 ± 0.3 ^a^	1.2 ± 0.6 ^b^	−0.2 ± 0.3 ^a^	−0.3 ± 0.4 ^a^
	b*	11 ± 2 ^ab^	14.4 ± 1.4 ^c^	12.8 ± 0.9 ^bc^	10.8± 1.4 ^a^
	∆E_1_*	-	3.9 ± 1.8 ^a^	3.6 ± 0.8 ^a^	3.1 ± 1.8 ^a^
Crust	L*	53 ± 5 ^a^	53 ± 3 ^a^	55 ± 2 ^ab^	58 ± 4 ^b^
	a*	8.98 ± 1.13 ^a^	10.8 ± 1.4 ^ab^	12 ± 2 ^b^	10 ± 3 ^ab^
	b*	20.8 ± 0.6 ^a^	26 ± 5 ^ab^	29 ± 5 ^b^	26 ± 6 ^ab^
	∆E_1_*	-	7 ± 3 ^a^	10 ± 3 ^a^	9 ± 3 ^a^

Data are presented as mean values ± standard deviation. Two independent dough batches were prepared per formulation, and three slices per batch were analyzed. Different superscript letters indicate significant differences (ANOVA, *p* < 0.05). L* indicates lightness (0 = black, 100 = white), a* indicates the red–green coordinate, and b* indicates the yellow–blue coordinate. RF: Reference Formulation. FA: Removal of monocalcium phosphate. FB: Removal of monocalcium phosphate and sodium bicarbonate. FC: Removal of monocalcium phosphate, sodium bicarbonate, and mono- and diglycerides of fatty acids.

**Table 5:** Mean values ± standard deviations of Texture Profile Analysis (TPA) parameters of the crumb and crust puncture force for different formulations at day 1 of storage.

Parameter	RF	FA	FB	FC
Crumb Hardness (N)	18 ± 4 ^a^	30 ± 4 ^c^	37.9 ± 1.2 ^d^	22 ± 4 ^b^
Crumb Cohesiveness	0.66 ± 0.03 ^ab^	0.68 ± 0.08 ^b^	0.62 ± 0.02 ^a^	0.66 ± 0.03 ^ab^
Crumb Springiness	0.95 ± 0.02 ^ab^	0.939 ± 0.013 ^ab^	0.97 ± 0.02 ^a^	0.98 ± 0.02 ^b^
Crumb Chewiness (N)	11 ± 2 ^a^	19.7 ± 4.0 ^c^	22.0 ± 0.8 ^c^	14.3 ± 1.9 ^b^
Crumb Gumminess (N)	12 ± 2 ^a^	21 ± 4 ^c^	23.5± 1.2 ^d^	14.8 ± 1.9 ^b^
Crust Puncture Force (N)	1.21 ± 0.16 ^ab^	1.58 ± 0.19 ^c^	1.4 ± 0.3 ^bc^	1.2 ± 0.3 ^a^

Data are presented as mean values ± standard deviation. Two independent dough batches were prepared per formulation, and four slices per batch were analyzed (eight measurements in total). Different superscript letters indicate significant differences (ANOVA, *p* < 0.05). RF: Reference Formulation. FA: Removal of monocalcium phosphate. FB: Removal of mono-calcium phosphate and sodium bicarbonate. FC: Removal of monocalcium phosphate, sodium bicarbonate, and mono- and diglycerides of fatty acids.

## Data Availability

The data presented in this study are available on request from the corresponding author due to industrial use.
